# Antioxidant Activity, Phenolic Profile, and Nephroprotective Potential of *Anastatica hierochuntica* Ethanolic and Aqueous Extracts against CCl_4_-Induced Nephrotoxicity in Rats

**DOI:** 10.3390/nu13092973

**Published:** 2021-08-26

**Authors:** Tariq I. Almundarij, Yousef M. Alharbi, Hassan A. Abdel-Rahman, Hassan Barakat

**Affiliations:** 1 Department of Veterinary Medicine, College of Agriculture and Veterinary Medicine, Qassim University, Buraydah 51452, Saudi Arabia; tmndrj@qu.edu.sa (T.I.A.); yhrby@qu.edu.sa (Y.M.A.); 2 Department of Physiology, Faculty of Veterinary Medicine, Sadat City University, Menofia 32897, Egypt; habdelrhman59@vet.usc.edu.eg; 3 Department of Food Science and Human Nutrition, College of Agriculture and Veterinary Medicine, Qassim University, Buraydah 51452, Saudi Arabia; 4 Food Technology Department, Faculty of Agriculture, Benha University, Moshtohor 13736, Qaliuobia, Egypt

**Keywords:** Kaff-e-Maryam, polyphenols, bioactivity, secondary metabolites, kidney markers, antioxidant enzymes, renal dysfunction

## Abstract

Kaff-e-Maryam (*Anastatica hierochuntica* L.) is extensively used to treat a range of health problems, most notably to ease childbirth and alleviate reproductive system-related disorders. This study aimed to evaluate the effect of *A. hierochuntica* ethanolic (KEE), and aqueous (KAE) extracts on CCl_4_-induced oxidative stress and nephrotoxicity in rats using the biochemical markers for renal functions and antioxidant status as well as histopathological examinations of kidney tissue. *A. hierochuntica* contained 67.49 mg GAE g^−1^ of total phenolic compounds (TPC), 3.51 µg g^−1^ of total carotenoids (TC), and 49.78 and 17.45 mg QE g^−1^ of total flavonoids (TF) and total flavonols (TFL), respectively. It resulted in 128.71 µmol of TE g^−1^ of DPPH-RSA and 141.92 µmol of TE g^−1^ of ABTS-RSA. *A. hierochuntica* presented superior antioxidant activity by inhibiting linoleic acid radicals and chelating oxidation metals. The HPLC analysis resulted in 9 and 21 phenolic acids and 6 and 2 flavonoids in KEE and KAE with a predominance of sinapic and syringic acids, respectively. Intramuscular injection of vit. E + Se and oral administration of KEE, KAE, and KEE + KAE at 250 mg kg^−1^ body weight significantly restored serum creatinine, urea, K, total protein, and albumin levels. Additionally, they reduced malondialdehyde (MOD), restored reduced-glutathione (GSH), and enhanced superoxide dismutase (SOD) levels. KEE, KAE, and KEE + KAE protected the kidneys from CCl_4_-nephrotoxicity as they mainly attenuated induced oxidative stress. Total nephroprotection was about 83.27%, 97.62%, and 78.85% for KEE, KAE, and KEE + KAE, respectively. Both vit. E + Se and *A. hierochuntica* extracts attenuated the histopathological alteration in CCl_4_-treated rats. In conclusion, *A. hierochuntica*, especially KAE, has the potential capability to restore oxidative stability and improve kidney function after CCl_4_ acute kidney injury better than KEE. Therefore, *A. hierochuntica* has the potential to be a useful therapeutic agent in the treatment of drug-induced nephrotoxicity.

## 1. Introduction

Kidney disease is the 9th leading cause of death with more than 1 in 7, that is, 15% of US adults or 37 million people, are estimated to have chronic kidney disease (CKD) [1]. Remarkably, the most common cause of CKD as recorded in 2015 is diabetes mellitus followed by high blood pressure and glomerulonephritis [2]. Other causes of CKD include idiopathic (often associated with small kidneys on renal ultrasound) [3]. Previously, CCl_4_ was used for metal degreasing, dry cleaning, fabric spotting, fire extinguisher fluids, and grain fumigation [4]. It causes severe disorders in the liver, lungs, and testes as well as in blood by generating active free radicals [5]. According to the findings of Ogeturk et al. [6], exposure to this solvent produces acute and chronic kidney damage. Free radical-induced lipid peroxidation is believed to be one of the primary causes of cell membrane damage, leading to a variety of pathological conditions [7]. The generation of reactive radicals has been implicated in CCl_4_-induced nephrotoxicity, which is involved in lipid peroxidation and accumulation of dysfunctional proteins, leading to injuries in kidneys [8]. Amazingly, traditional uses of medicinal plants have grown in recent years, and numerous investigations have confirmed their therapeutic role against several illnesses [9,10,11,12].

Kaff-e-Maryam (*Anastatica hierochuntica*) is a desert medicinal herb that belongs to the Brassicaceae family. It grows in various regions over the world, especially in Arab countries (e.g., Saudi Arabia, Egypt, Oman, Libya, Iraq, the United Arab Emirates, Kuwait) as well as some Asian, European, and African countries. *A. hierochuntica* is believed to have superior medical potentials and is preferably consumed for various medical conditions [13]. It is mainly used to ease the process of childbirth and for treating reproductive system-related disorders and metabolic disorders, mainly diabetes mellitus [14]. It is used as an analgesic and as a treatment for epilepsy, gastrointestinal disorders, arthritis, bronchial asthma, mouth ulcers, malaria, and mental depression [15,16,17]. *A. hierochuntica* contains significant amounts of minerals, such as Mg, Ca, Mn, Fe, Cu, and Zn, which are comparable to or greater than those of many herbal plants, which may give metals chelating properties [18]. Yoshikawa et al. [19] concluded that the presence of flavanones, such as Anastatin A and B, correlates with potent hepatoprotective effects by inhibiting *D*-galactosamine-induced cytotoxicity in primary cultured mouse hepatocytes. The production of reactive oxygen species (ROS) and cytokines such as tumor necrosis factor and interleukin-1 by Kupffer cells in the liver contribute to hepatocyte destruction in D-galactosamine hepatotoxicity [20]. The antioxidant and anti-inflammatory properties of *A. hierochuntica* components may help to reduce *D*-galactosamine-induced hepatotoxicity [21]. *A. hierochuntica* can afford extract-depending protection against CCl_4_-hepatotoxicity [22]. However, despite the literature showing promising potentialities related to the use of *A. hierochuntica*, the nephroprotective potential of *A. hierochuntica* ethanolic (KEE) and aqueous (KAE) extracts needs to be carefully examined. Moreover, the literature review mainly highlighted the hepatoprotective efficiency of *A. hierochuntica*, but the nephroprotective potential has not been studied so far, thus motivating this work. Therefore, the current study aims to observe the changes in the antioxidative defense enzymes, detect the alterations of renal microscopy after CCl_4_ administration in rats, and investigate the possible protective effects of *A. hierochuntica* extracts against CCl_4_-induced renal damage.

## 2. Materials and Methods

### 2.1. Sample Preparation

A sample of the Kaff-e-Maryam (*A. hierochuntica* L.) plant was purchased from a native market in Buraydah city, Qassim region, Saudi Arabia. The plant material was authenticated by the Department of Plant Production and Protection, College of Agriculture and Veterinary Medicine, Qassim University, Saudi Arabia. The sample was washed with clean tap water to remove sand and dirt from the leaves and then air-dried plant material (at 28 ± 1 °C for 48 h.) was mechanically powdered and kept in opaque polyethylene bags at 4 ± 1 °C until use.

### 2.2. Preparation of Ethanolic and Aqueous Extracts

Approximately 200 g of dried *A. hierochuntica* were extracted with 300 mL 70% ethanol in a Soxhlet extractor to prepare ethanolic extraction (KEE). The extract was concentrated by a rotary evaporator at 40 °C to evaporate the remaining solvent, then to dryness under an N_2_ stream. The aqueous extraction (KAE) was carried out as described by Asuzu [23] with minor modifications. Two hundred grams of dried plant material were added to 500 mL of hot sterile distilled water. The mixture was then shaken well and allowed to stand for 1 h. Then a reflux condenser was attached to the flask and then heated until boiling gently for 10 min, cooled, shaken well, and filtered through Whatman No. 1 filter paper. The filtrate was evaporated by a rotary evaporator, then to dryness under an N_2_ stream. The alcoholic and aqueous extracts (250 mg mL^−1^) were freshly formulated in distilled water to be used for oral administration.

### 2.3. Total Phenolic Content (TPC)

The TPC content of *A. hierochuntica* was determined according to the adapted method by Bettaieb et al. [24]. The results were compared to a plotted gallic acid (GA) standard curve made in the range of 50–500 mg mL^−1^ (*R*^2^ = 0.99), and the TPC was calculated as mg of gallic acid equivalent (GAE) per gram of *A. hierochuntica* (mg of GAE g^−1^).

### 2.4. Total Carotenoids (TC), Total Flavonoids (TF), and Total Flavonols (TFL)

As reported by Al-Qabba et al. [10], 5 g of *A. hierochuntica* was extracted repeatedly with acetone and petroleum ether mixture (1:1, *v*/*v*). Total carotenoids (TC) content was spectrophotometrically determined at 451 nm. TC was expressed as mg g^−1^ dw. The TF content of *A. hierochuntica* was assayed according to described protocol by Mohdaly et al. [25]. The TF content was calculated as mg quercetin equivalent (QE) per 100 g^−1^ dw. In the same context, the TFL content was carried out [26]. The absorbance at 440 nm was recorded, and TFL was calculated as mg quercetin equivalent (QE) per 100 g^−1^ dw.

### 2.5. Antioxidant Capacity Determination

DPPH radical scavenging assay: The RSA was tested spectrophotometrically depending on bleaching of DPPH purple-colored solution according to an altered technique by Lu et al. [27]. The antiradical capacity was presented as µmol of Trolox equivalents (TE) per gram of *A. hierochuntica* (µmoL TE g^−1^). ABTS radical scavenging activity: The RSA of *A. hierochuntica* against ABTS radicals was tested by the adapted method of Lu et al. [27]. The results were expressed as µmoL TE g^−1^. β-carotene–linoleic acid bleaching assay: The antioxidant percentage of *A. hierochuntica* was assessed in terms of β-carotene bleaching in comparison to butylated hydroxyanisole (BHA) applying an adapted spectrophotometric protocol designated by Koleva et al. [28]; the results were given as BHA-related percentage. Chelating action of *A. hierochuntica* on ferrous ions: The chelating activity of *A. hierochuntica* was measured as protocoled by Zhao et al. [29]. The inhibition % of ferrozine–Fe^2+^ complex creation as metal chelating action was measured and presented as mg mL^−1^ when ethylenediaminetetraacetic acid (EDTA) as a positive control was used.

### 2.6. Polyphenolic Compound Fractionation of A. hierochuntica Aqueous and Ethanolic Extracts

Determination of polyphenols from ethanolic and aqueous extracts was performed by an HP1100 (Agilent Technologies, Palo Alto, CA, USA) HPLC system equipped with an auto-sampler, quaternary pump, and diode array detector (Hewlett Packard 1050) using a column (Alltima C18 150 × 4.6 mm, 5 µm) with a 5 mm Altima C18 guard column (Alltech, Nettetal, Germany) according to Goupy et al. [30]. The solvent system used was a gradient of A (acetic acid 2.5%), B (acetic acid 8%), and C (acetonitrile). The solvent flow rate was 1 mL min^−1^, and separation was performed at 35 °C. The injected volume was 10 µL. Phenolic compounds were assayed by external standard calibration and expressed as mg g^−1^ dw of equivalents (+)-catechin for flavan-3-ols and equivalent coumarin for polar aromatic compounds. A variability of 8% was determined on five extractions of phenolics from the same sample. All values were the mean of duplicate injections. Polyphenols and their derivatives were identified and quantified at 280 and 320 nm, while flavonoids were identified at 360 nm.

### 2.7. Experimental Design 

All experiments were approved by the Institutional Animal Ethics Committee (IAEC) of QU (No. 15-4-2017), KSA, which is regulated by the Purpose of the Control and the Supervision of Experiments on Animals (CPCSEA) Committee under the National Committee of BioEthics (NCBE), Implementing Regulations of the Law of Ethics of Research on Living Creatures. A total of 36 male albino rats were used in the current study and divided into 6 groups of 6 animals each and treated as follows: Group I (Control) received an intraperitoneal injection (i.p.) of olive oil (1.0 mL kg^−1^ twice a week) and 0.5 mL distilled water orally/daily for 21 successive days. Group II received i.p. injection of a fresh mixture of equal volumes of CCl_4_ and olive oil (at a dose of 1.0 mL kg^−1^ twice a week) and 0.5 mL distilled water orally/daily according to Al-Qabba et al. [10] with minor modifications. Group III (reference group) received an intramuscular injection (i.m.) of 50 mg kg^−1^ vit. E + Se (Selepherol, Vetoquinol Co., Magny-Vernois, France) twice a week, according to Asuku et al. [31] and El-Desoky et al. [32], and 0.5 mL distilled water orally/daily. Group IV served as a test and received 250 mg kg^−1^ of KEE orally/daily along with CCl_4_ i.p. twice a week. Group V received 250 mg kg^−1^ of KAE orally/daily along with CCl_4_ i.p. twice a week. Group VI received 250 mg kg^−1^ of KEE + KAE (1:1) orally/daily along with CCl_4_ i.p. twice a week. Twenty-four hours after the last treatment (day 21), the rats were anesthetized by the mixture (alcohol:chloroform:ether, 1:2:3). Blood samples from heart puncture were collected for all animals, and serum was separated by centrifugation at 4000 rpm for 10 min and kept at −20 °C for biochemical examination.

#### 2.7.1. Kidney Biochemical Analysis 

Serum creatinine, urea, total protein, and albumin concentrations were determined by automated spectrophotometric methods (BM/Hitachi autoanalyzer-911; Boehringer Mannheim, Germany) according to the instructions of the manufacturer. Potassium levels were determined by flame photometry at 766 nm.

#### 2.7.2. Estimation of Renal Antioxidant Activity 

After the collection of blood samples, animals of all groups were sacrificed; right kidneys were rapidly isolated and rinsed with ice-cold saline. The tissue was then clipped, rinsed in cold saline, blotted dry, and placed on ice immediately. Using an electrical tissue homogenizer, portions of the tissue (1.0 g) were weighed and homogenized with 9 volumes of ice-cold 0.05 M phosphate buffer at pH 7.4. Cell debris was removed by centrifugation at 12,000 rpm (4 °C) for 20 min to collect supernatants for determination of malondialdehyde (MDA) concentration [33], superoxide dismutase (SOD) activity [34], and reduced glutathione (GSH) content [35]. Protein concentration in kidney homogenate was determined using the Bradford method [36].

#### 2.7.3. Nephroprotection Percentage

The nephroprotection (F) percentages of vit. E + Se, KEE, KAE, and KEE + KAE were calculated for each biochemical parameter separately according to Wakchaure et al. [37] using the following equation:

(1) $$F\%=[1-\frac{\left(T-N\right)}{\left(C-N\right)}]\times 100$$

where T = mean value of treatment group, C = mean value of the positive control group, and N= mean value of the negative control group. Moreover, the total nephroprotection percentage (TFP %) was compared to vit. E + Se as follows:

(2) $$\mathrm{TFP}\%=\frac{\mathrm{Sum}\;\mathrm{of}\;F\%\;\mathrm{of}\;\mathrm{the}\;\mathrm{biochemical}\;\mathrm{parameters}\;\mathrm{of}\;\mathrm{each}\;\mathrm{extract}}{\mathrm{sum}\;\mathrm{of}\;F\%\;\mathrm{of}\;\mathrm{the}\;\mathrm{biochemical}\;\mathrm{parameters}\;\mathrm{of}\;\mathrm{vit}.E+\mathrm{Se}}\times 100$$

#### 2.7.4. Histopathological Studies

Autopsy samples were collected from the left kidney of separate groups of rats and fixed in 10% formalin saline for 24 h. Washing with tap water was followed by dehydration with serial dilutions of alcohol (methyl, ethyl, and absolute ethyl). Specimens were cleaned in xylene and embedded in paraffin for 24 h at 56 °C in a hot air oven. Paraffin bees wax tissue blocks were prepared for sectioning at 4-micron thickness using a sled microtome. Tissue slices were collected on glass slides, deparaffinized, and stained with hematoxylin and eosin for regular inspection under a light electric microscope [38].

### 2.8. Statistical Analysis

The results are shown as mean ± standard error (SE). The significance of differences between means in various groups was examined using a one-way analysis of variance (ANOVA) followed by Duncan’s test, and a *p*-value among means was given at the *p* < 0.05 level [39].

## 3. Results

### 3.1. Phytochemicals and Antioxidant Capacity of A. hierochuntica 

The quantitative analysis of *A. hierochuntica* phytochemicals and related antioxidant activities using DPPH and ABTS radical scavenging, β-carotene–linoleic acid bleaching activities, and chelating ability (CA) were performed. As can clearly be seen in Table 1, TPC content was 67.49 mg GAE g^−1^. The TC content was 3.51 µg g^−1^. The TF and TL contents were 49.78 and 17.45 mg QE g^−1^, respectively. Moreover, DPPH-RSA and ABTS-RSA were used to measure the progression of antioxidant activities. Results indicated 128.71 µmol of TE g^−1^ and 141.92 µmol of TE g^−1^ for DPPH-RSA and ABTS-RSA, respectively. Additionally, the antioxidant activity (AOA) of *A. hierochuntica* is presented in Table 1. The inhibition percentage of linoleic acid radicals was calculated as 45.74% comparing to BHA using β-Carotene bleaching (β-CB) assay. Furthermore, evaluation of the metal-chelating activity revealed 42.89 mg g^−1^, which seems to be proficient in interfering with Fe^2+^–ferrozine complex formation, indicating its capability to chelate oxidation metals. 

### 3.2. Quantification of A. hierochuntica Phenolic Compounds 

The quantitative analysis of phenolic compounds for KEE and KAE by HPLC analysis was carried out, and data are tabulated in Table 2. Nine separated phenolic acids and six flavonoids were identified in detectable amounts from the KEE of *A. hierochuntica*. The most abundant phenolic acids were hydroxycinnamic acids such as sinapic acid (28.704 mg 100 g^−1^) followed by caffeic acid (6.621 mg 100 g^−1^), rosmarinic acid (2.884 mg 100 g^−1^), ferulic acid (1.854 mg 100 g^−1^), and cinnamic acid (0.094 mg 100 g^−1^); and hydroxy-benzoic acids such as *p*-hydroxybenzoic acid (3.440 mg 100 g^−1^), protocatechuic acid (1.811 mg 100 g^−1^), vanillic acid (3.326 mg 100 g^−1^), and syringic acid (1.083 mg 100 g^−1^). Flavonoids such as myricetin (16.269 mg 100 g^−1^), *D*-catechin (2.410 mg 100 g^−1^), kaempferol (0.434 mg 100 g^−1^), rutin (0.539 mg 100 g^−1^), apigenin-7-glucoside (0.192 mg 100 g^−1^), and quercetin (0.184 mg 100 g^−1^) in valuable amounts were detected. The phenolic compounds in KAE of *A. hierochuntica* were also determined, and data are tabulated in Table 2. Syringic acid was recorded as the highest phenolic acid among the 21 identified phenolics. Catechol and pyrogallol were 2.526 and 1.589 mg 100 g^−1^, respectively. Data indicated that some phenolic acids such as caffeic, catechin, chlorogenic, epicatechin, *e*-vanillic, *p*-hydroxybenzoic, and protocatechuic acids were detected in the moderate amounts of 0.725, 0.256, 0.136, 0.193, 0.443, 0.223, and 0.454 mg 100 g^−1^, respectively. In the same context, low amounts of 3,4,5-trimethoxycinnamic, 4-aminobenzoic, benzoic, cinnamic, coumarin, ellagic, ferulic, gallic, iso-ferulic, α-coumaric, *p*-coumaric, and salicylic acids were quantified after being identified. Epicatechin and *D*-catechin as flavonoids were quantified in KAE of *A. hierochuntica* as well.

### 3.3. Serum Creatinine, Urea, K, Total Protein, and Albumin Levels

CCl_4_ injection substantially raised serum creatinine, urea, and k levels in GII rats when compared to control rats (GI). Conversely, total protein and albumin levels were significantly decreased in CCl_4_-treated rats (Table 3). Vit. E + Se and *A. hierochuntica* extracts (G III, IV, V, and VI) substantially reduced the alterations in creatinine and urea caused by CCl_4_ injection, while they increased albumin and total proteins to be close to normal values in GI (Table 3). Serum k level was markedly increased in CCl_4_-treated rats (GII) when compared to GI (Table 3). The injection of vit. E + Se and administration of *A. hierochuntica* alcoholic and aqueous extracts (G IV, V, and VI) was also positively improve the k level when compared to GI (Table 3). 

### 3.4. Renal Antioxidant Biomarkers 

As shown in Table 4, administration of CCl_4_ significantly reduced SOD and GSH levels and increased the MDA level in GII kidney homogenate tissue. However, when compared to GI, rats treated with both vit. E + Se and *A. hierochuntica* extracts (GIII, VI, V, and VI) exhibited a substantial improvement in the activity of antioxidant enzymes SOD and GSH, as well as a reduction in MDA levels (Table 4). *A. hierochuntica* alcoholic extract (GIV) outperformed *A. hierochuntica* aqueous extract (GV) and combined *A. hierochuntica* alcoholic and aqueous extracts in attenuating antioxidant levels, and combating the autoxidation process resulted in low MDA levels when compared to GI.

### 3.5. Nephroprotection Percentage

The nephroprotection percentage (relative to the negative control (GI) and positive (GII) groups) of kidney functions such as creatinine, urea, k, TP, and albumin as well as antioxidant activities in kidney homogenate (MDA, SOD, GSH) is illustrated in Table 5. The nephroprotection % recorded the highest value as creatinine, urea, k in GIII, TP, and albumin in GV, MDA, and GSH in GIII and SOD in GV (Table 5). The total nephroprotection % relative to vit. E + Se treatment registered maximum levels in the KAE treated group (GV, 97.62%), then KEE (GIV, 83.27%), and then KEE + KAE (GVI, 78.85%), as revealed in Table 5.

### 3.6. Effects of A. hierochuntica Extracts on Renal Histoarchitecture

**Figure 1:**
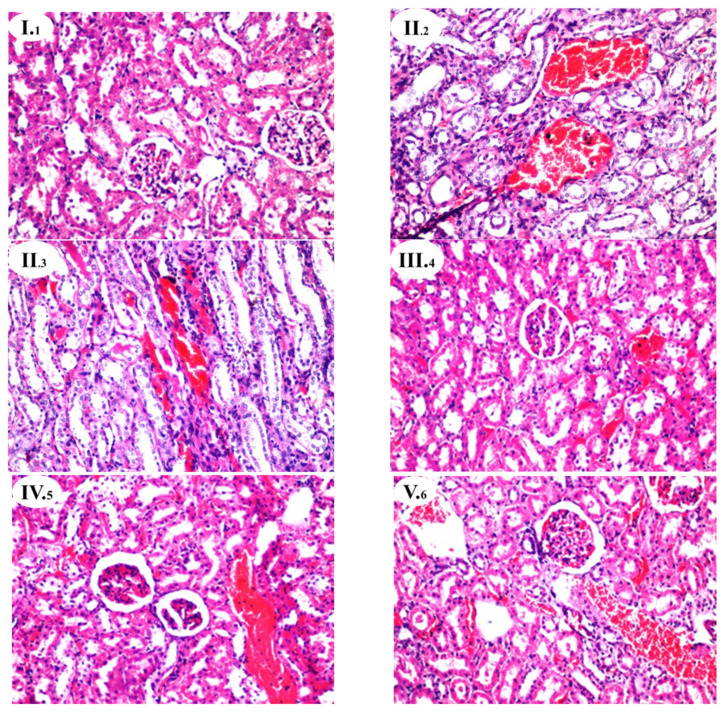

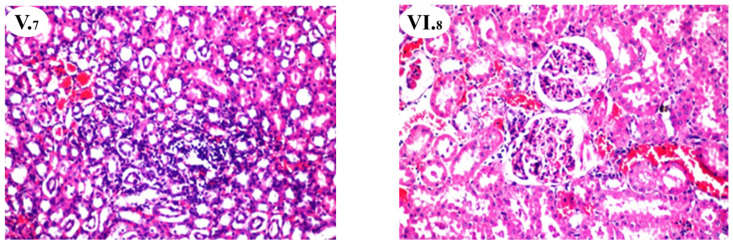
Histopathological findings of rat kidney protection by *A. hierochuntica* extracts (Hematoxylin-Eosin (HE), 200X). Histopathological nephrotoxicity induction. GI: Histopathological structure of control group with normal histological structure of the glomeruli and tubules of rat kidney (**I._1_**). GII: Kidney of a rat receiving only CCl_4_ showing focal inflammatory cell infiltration in between the tubules at the cortex (**II._2_**). There was congestion in the blood vessels between the tubules, and multiple eosinophilic casts formed in the lumen of some tubules at the cortex. The corticomedullary portion showed focal hemorrhage and hemolysis in between the degenerated tubules (**II._3_**). GIII: Kidney of a rat receiving CCl_4_ + vit. E + Se showing congestion in the blood vessels in between the tubules at the cortex (**III._4_**). GIV: Kidney of a rat receiving CCl_4_ + KEE viewing congestion in the cortical blood vessels (**IV._5_**). GV: rat kidney receiving CCl_4_ + KAE showing congestion in the cortical blood vessels (**V._6_**). The corticomedullary junction showed focal inflammatory cells infiltration with eosinophilic renal casts formation in the lumen of some tubules (**V._7_**). GVI: kidney of rats receiving CCl_4_ + KEE + KAE showing congestion in the cortical blood vessels and glomeruli (**VI._8_**).

The results of the biochemical investigations were supported by histopathological examination. Table 6 and Figure 1 show the degree of histological changes in the underlying structure of the rat’s kidneys in various experimental groups treated with *A. hierochuntica* extracts. In the current investigation, the kidney of the control group (GI) was found to have a normal histological structure (Figure 1(I._1_)). The histoarchitecture of the CCl_4_-treated rats (GII) showed focal inflammatory cell infiltration (++) between the tubules at the cortex, congestion (++) of blood vessels between the tubules (Figure 1(II._2_)), multiple eosinophilic cast (++) formations in the lumen of some tubules, and focal hemorrhage (++) between the degenerated tubules at the corticomedullary portion (Figure 1(II._3_), Table 6). In GIII, injecting vit. E + Se, administrating the alcoholic extract of *A. hierochuntica* (GIV), aqueous extract of *A. hierochuntica* (GV), and a combination of *A. hierochuntica* extracts (GVI) attenuated the cytotoxic effects of CCl_4_ and recorded mild (+) to moderate (++) congestion in the blood vessels among tubules at the cortex (Figure 1(III._4_–VI._8_), Table 6) with well-developed Bowman’s capsule with glomerulus and convoluted tubules enlarged. Additionally, the aqueous extract of *A. hierochuntica* (GV) recorded focal inflammatory cell infiltration (++) at the corticomedullary portion (Figure 1(V._7_), Table 6).

## 4. Discussion

Phytochemicals are mostly effective free radical scavengers and are considered plant-based superior antioxidant agents. Polyphenolic substances are thought to have anti-carcinogenic and anti-mutagenic properties in humans [40]. A valuable TPC content in *A. hierochuntica* was slightly higher than that obtained by Mohamed et al. [21] as 51.97 mg GAE g^−1^ in *A. hierochuntica* herb and by AlGamdi et al. [41], who found 4 mmol L^−1^ GAE in *A. hierochuntica* seeds. Recently, Zin et al. [42] indicated the presence of tannins in *A. hierochuntica* as a bioactive compound and recommended its bioactivity, which needed to be deeply investigated. The β-carotene content, as a part of total carotenoids, was 2.27 μg g^−1^ as mentioned by Mohamed et al. [21], and even current results presented total carotenoids as 3.51 μg g^−1^. Similar findings in flavonoid and flavonol contents have been indicated by Mohamed et al. [21]. Biologically active components, such as phenolic compounds, present antioxidant activity as breakdowns of lipid oxidation chain reactions by giving hydrogen to active free radicals. This scavenging potential of phenolics to inhibit radicals was elucidated by their phenolic hydroxyl groups [8,10,22]. This phenolic acid has been described as an effective antioxidant component, including hydrogen peroxide, hydroxyl radical, and superoxide anion [43]. *A. hierochuntica* metal chelating activity seems to be proficient in interfering with “Fe^2+^–ferrozine” complex construction, suggesting its ability to capture “ferrous” ions before “ferrozine”. A positive relationship between an increase in their contents of phenolic compounds is directly indicated with their antioxidant capacity [42]. Andjelković et al. [44] established the activity of numerous phenolic acids to form complexes with metals. The valuable TPC and relevant antioxidant activities using different measuring approaches give a clear plate from and confirm the bioactivity of *A. hierochuntica* as a medicinal herb for food or beverage applications.

Biologically active components, such as phenolic compounds, present antioxidant activity as breakdowns of lipid oxidation chain reactions by donating hydrogen to active free radicals [45]. This scavenging potential of phenolics to inhibit radicals was elucidated by their phenolic hydroxyl groups [46]. This phenolic acid has been described as an effective antioxidant component, including hydrogen peroxide, hydroxyl radical, and superoxide anion [43,45,47]. The identified and quantified compounds by HPLC in KAE of *A. hierochuntica* were higher than the number of identified compounds in KEE, but identified compounds in KEE of *A. hierochuntica* were presented in higher amounts [22]. The results reflect that the consuming *A. hierochuntica* could present many components in both polar and non-polar forms. These results are similarly presented by AlGamdi et al. [41] as they identified and quantified 20 polyphenolic compounds in seeds of *A. hirerochuntica*. The extract contained chlorogenic acids and hydroxybenzoic acids, but the main components were flavone *C*-glycosides, *C*-diglycosides, *O*-glycosides, and *O*-glycoside-*C*-glycosides occurring predominantly as luteolin conjugates. In addition, 14 of the 20 compounds in *A. hierochuntica* extract exhibited antioxidant activity using an HPLC-on-line antioxidant detection system [41]. Interestingly, current data confirmed that *A. hierochuntica* is rich in phytochemicals compounds and is a good source of natural antioxidants with potential health benefits, as has been scarcely highlighted before in seeds [41]. Hence, tea prepared from the whole plant powder is the traditional form of consumption; data illustrated new identified bioactive compounds in KEE and KAE of *A. hirerochuntica*, which differed from those found in AlGamdi et al. [41]

In numerous studies, CCl_4_-induced nephrotoxicity is utilized as a model system for testing the nephroprotective effect of plant extracts/drugs [48,49]. The current study looked at the effect of *A. hierochuntica* extracts on CCl_4_-induced kidney damage, as well as its nephroprotection and antioxidant potential in rats. In the current study, the CCl_4_ treatment (GII) group significantly increased creatinine, urea, and k levels and decreased total protein and albumin concentrations when compared to GI. This might be because CCl_4_ intoxication is a major source of free radical production in numerous organs, including the liver, kidneys, lungs, brain, and blood [50]. It has also been observed that following CCl_4_ injection in rats, the concentration of CCl_4_ is distributed more evenly in the kidneys than in the liver [51], since the kidney has a high affinity for CCl_4_ and contains cytochrome P450, predominantly in the cortex. The most common free radicals from CCl_4_ are trichloromethyl radical (CCl_3_^•^) and trichloromethyl peroxyl radical (CCl_3_O_2_^•^) [52]. These radicals attach to an intracellular protein, cell membrane lipids, and DNA, causing protein denaturation, lipid peroxidation, and oxidative DNA damage that leads to cell death [53]. In contrast, treating CCl_4_-rats with vit. E + Se (GIII) and *A. hierochuntica* extracts (GVI: VI) efficiently attenuated these rises in creatinine and urea levels as well as increased serum albumin and total proteins to be very close to their levels in GI. This may be due to the antioxidant properties and rich phenolic content of *A. hierochuntica* extracts and antioxidant capacity and chelating activity of vit. E + Se, which scavenges free radicals thereby inhibiting the renal damage. Phytochemicals are the most highly effective free radical scavengers and are considered superior antioxidant agents from plants [54]. The most abundant phenolic compounds were hydroxycinnamic acids, such as sinapic acid, among the nine identified phenolic compounds in KEE, while syringic acid was the highest phenolic acid among the 21 identified phenolic acids in KAE. Six flavonoids were identified in KEE and two in KAE using HPLC analysis [55]. Furthermore, as an antioxidant, vit. E is believed to protect tissues from harm caused by reactive oxygen metabolites. Selenium is also well recognized to be an essential trace mineral for human health, shielding cells from the damaging effects of free radicals [22].

In the current study, CCl_4_ administration markedly decreased GSH and SOD and increased MDA levels in kidney homogenates relative to GI. Vit. E + Se and *A. hierochuntica* extracts ameliorated the diverse effects of CCl_4_ by restoring the altered activity of antioxidant agents such as SOD and GSH and may deactivate the process of producing the MDA, as was recently reported [15,21,40,41]. GSH is a non-enzymatic antioxidant that is found in all mammalian cells. With its oxidized form, GSSG, GSH acts as a cofactor for numerous detoxifying enzymes (GPx, GST, and others) against oxidative stress and maintains cellular redox balance [47]. This finding is in accordance with those of Khan and Siddique [56] and Makni et al. [57], who reported that CCl_4_ decreased the GSH level in rat kidneys. Treatment with vit. E + Se and *A. hierochuntica* extracts showed protection against reduction in GSH level triggered by CCl_4_. In the same context, SOD catalyzes the dismutation of two molecules of superoxide anion (*O_2_) to hydrogen peroxide (H_2_O_2_) and molecular oxygen (O_2_), consequently rendering the potentially harmful superoxide anion less hazardous [58,59]. CCl_4_ intoxication alters the gene expression level by depleting renal SOD [60]. A decrease in SOD activity is a sensitive index of cellular damage. Our tested *A. hierochuntica* extracts ameliorated renal toxicity by alleviating the level of SOD. It participates in various enzymatic processes to reduce the concentration of detoxification reactions [61]. MDA is the first product of lipid peroxidation and is one of the important markers of oxidative stress. *A. hierochuntica* extracts diminished the increase in MDA levels and restored total antioxidant power in the CCl_4_-treated rat kidneys. These protective effects may be due to the powerful antioxidative activity of *A. hierochuntica* extracts [15,21,40,41]. These results also suggest that *A. hierochuntica* extracts may attenuate oxidative stress by decreasing levels of lipid peroxide in CCl_4_-exposed rat kidneys and prevent renal damage. These results agreed with the results of the antioxidative activities of Zn on CCl_4_-induced acute nephrotoxicity [62,63].

*A. hirerochuntica* extracts presented valuable nephroprotection capacity regarding kidney function tests (creatinine, urea, K, TP, and albumin) and kidney homogenate antioxidant activities (GSH, SOD, MDA) in GIV, V, and IV, respectively. The total nephroprotection % relative to vit. E + Se treatment registered maximum levels in the KAE treated group (GV, 97.62%), then KEE (GIV, 83.27%), and then KEE + KAE (GVI, 78.85%), respectively, in descending order. This may be due to differences in quantity and quality of phenolic and antioxidant contents of *A. hirerochuntica* extracts, which have a relation to antioxidant capacity [15,19,22,40,42].

The histopathological findings in kidneys are consistent with the biochemical estimations of the experimental groups investigated. CCl_4_ administration (GII) caused a glomerular and tubular lesion with vasocongestion in the kidneys. Dogukan et al. [64] discovered a similar histological pattern in rat renal tissue in response to prolonged CCl_4_ treatment. It is also considered that histological changes are caused by functional overloading of nephrons, which leads to renal dysfunction [65], and/or are due to the destruction of tissue provoked as a consequence of free radical generation via CCl_4_ metabolism [56,66]. The effect of vit. E + Se and *A. hierochuntica* extracts to repair and restore kidneys destruction effects of CCl_4_ were notable. This may be because vit. E + Se (as a potent antioxidant) acts on ROS induced by CCl_4_ [67]. *A. hierochuntica* extracts suppress CCl_4_-induced acute nephrotoxicity due to the antioxidative role and free radical scavenging properties of the phenolic compounds present in *A. hirerochuntica* extracts [22]. Our findings are consistent with those of other researchers who have shown that various plant derivatives have pharmacological effects by eliminating CCl_4_ abuses and restoring to normality [6].

## 5. Conclusions

Results of this study clearly demonstrated that *A. hierochuntica* plant is rich in polar and nonpolar phenolic compounds with a superior antioxidant capacity, which is directly related to the phytochemical content. *A. hierochuntica* (particularly aqueous extract) protects rats against CCl_4_-induced oxidative stress and acute kidney injury, as evidenced by a significant drop in MDA levels and increased GSH and SOD activity, as well as the cessation of biochemical and histological alterations in the kidneys. The protective efficacy might arise from the antioxidant and free radical scavenging properties of the phenolic compounds present in the *A. hierochuntica* extracts. These characteristics help to explain the plant’s medicinal efficacy as a herbal medication. More research is needed to completely describe the active principles in *A. hierochuntica*, and this study is meant to stimulate more comprehensive related research to offer sufficient data and recommendations for defining its mechanisms and safe doses.

## Figures and Tables

**Table 1:** Total phenolic content, total carotenoids, total flavonoids, total flavonols, and relative potential antioxidant activities of *A. hierochuntica* (mean ± SE), *n* = 6.

Item	*A. hierochuntica*
TPC (mg GAE g^−1^)	67.49 ± 3.33
TC (µg g^−1^)	3.51 ± 0.91
TF (mg QE g^−1^)	49.78 ± 2.62
TFL (mg QE g^−1^)	17.45 ± 0.83
DPPH (µmol of TE g^−1^)	128.71 ± 3.55
ABTS (µmol of TE g^−1^)	141.92 ± 4.67
β-CB * (RAA) %	45.74 ± 4.80
CA (mg g^−1^)	42.89 ± 2.11

Note: *: relatively calculated based on BHA as 100%, RAA: relative antioxidant activity.

**Table 2:** Quantitative analysis of phenolic compounds from KEE and KAE of *A. hierochuntica* by HPLC-DAD.

Item	No.	Compound	Ethanolic Extract (KEE) (mg 100 g^−1^)	Aqueous Extract (KAE) (mg 100 g^−1^)
Phenolic acids	1	3,4,5-trimethoxycinnamic acid	-	0.042
	2	4-Aminobenzoic acid	-	0.012
	3	Benzoic acid	-	0.005
	4	Caffeic acid	6.621	0.725
	5	Catechol	-	2.526
	6	Chlorogenic acid	-	0.136
	7	Cinnamic acid	0.094	0.001
	8	Coumarin	-	0.036
	9	Ellagic acid	-	0.039
	10	*e*-Vanillic acid	-	0.443
	11	Ferulic acid	1.854	0.037
	12	Gallic acid	-	0.041
	13	Iso-ferulic acid	-	0.005
	14	*α*-Coumaric acid	-	0.039
	15	*p*-Coumaric acid	-	0.009
	16	*p*-Hydroxybenzoic acid	3.440	0.223
	17	Protocatechuic acid	1.811	0.454
	18	Pyrogallol	-	1.589
	19	Rosmarinic acid	2.884	-
	20	Salicylic acid	-	0.089
	21	Sinapic acid	28.704	-
	22	Syringic acid	1.083	1.959
	23	Vanillic acid	3.326	1.406
Flavonoids	1	Apigenin-7-glucoside	0.192	-
	2	*D*-Catechin	2.410	0.256
	3	Epicatechin	-	0.193
	4	Kaempferol	0.434	-
	5	Myricetin	1.627	-
	6	Quercetin	0.184	-
	7	Rutin	0.539	-

Notes: KEE: *Anastatica hierochuntica* ethanolic extract; KAE: *Anastatica hierochuntica* aaqueous extract.

**Table 3:** Effect of oral administration of *A. hierochuntica* extracts on biochemical kidney markers in CCl_4_-induced toxicity in rats (mean ± SE), *n* = 6.

Kidney Functions	Experimental Groups					
	GI	GII	GIII	GIV	GV	GVI
Creatinine (mg dL^−1^)	0.88 ± 0.09 ^a^	1.30 ± 0.11 ^b^	0.87 ± 0.11 ^a^	0.99 ± 0.07 ^a^	1.08 ± 0.03 ^a^	0.91 ± 0.11 ^a^
Urea (mg dL^−1^)	77.59 ± 2.60 ^a^	117.00 ± 3.98 ^b^	77.53 ± 10.11 ^a^	73.60 ± 5.35 ^a^	78.65 ± 12.69 ^a^	70.33 ± 8.37 ^a^
K (mEq L^−1^)	4.18 ± 0.21 ^a^	5.55 ± 0.68 ^bc^	4.57 ± 0.23 ^ab^	4.78 ± 0.21 ^b^	5.00 ± 0.21 ^b^	5.48 ± 0.23 ^c^
Total proteins (g dL^−1^)	8.71 ± 0.92 ^c^	5.04 ± 0.36 ^a^	7.54 ± 0.45 ^b^	7.89 ± 0.44 ^bc^	8.59 ± 0.18 ^c^	5.89 ± 1.43 ^ab^
Albumin (g dL^−1^)	3.91 ± 0.13 ^b^	3.28 ± 0.09 ^a^	3.79 ± 0.31 ^b^	3.68 ± 0.16 ^b^	4.34 ± 0.17 ^c^	3.71 ± 0.14 ^b^

GI: control negative group, GII: control positive group received CCl_4_ (i.p.), GIII: CCl_4_-rats received 50 mg kg^−1^ vit. E + Se twice a week (i.m.), GIV: CCl_4_-rats received KEE as 250 mg kg^−1^ per oral (p.o.) daily, GV: CCl_4_-rats received KAE as 250 mg kg^−1^ (p.o.) daily and GVI: CCl_4_-rats received KEE + KAE (1:1) as 250 mg kg^−1^ (p.o.) daily. ^a–c^: values with the same superscript letter in the same raw are not significantly different at *p* ≤ 0.05.

**Table 4:** Effects of oral administration of *A. hierochuntica* extracts on kidney oxidative damage in CCl_4_-induced toxicity in rats (mean ± SE), *n* = 6.

Oxidative Stress Markers	Experimental Groups					
	GI	GII	GIII	GIV	GV	GVI
MDA nmol/mg protein	131.68 ± 10.83 ^a^	308.58 ± 18.27 ^c^	125.01 ± 12.40 ^a^	151.46 ± 9.01 ^a^	242.06 ± 40.81 ^b^	285.75 ± 20.47 ^b^
SOD nmol/mg protein	22.66 ± 0.54 ^c^	11.47 ± 2.01 ^a^	18.16 ± 0.99 ^b^	16.32 ± 1.51 ^b^	21.98 ± 0.97 ^c^	20.16 ± 1.87 ^bc^
GSH nmol/mg protein	3.64 ± 0.15 ^b^	2.42 ± 0.25 ^a^	3.83 ± 0.55 ^b^	3.40 ± 0.15 ^b^	3.48 ± 0.18 ^b^	3.82 ± 0.26 ^b^

MDA: malondialdehyde, SOD = superoxide dismutase, GSH: reduced glutathione, GI: control negative group, GII: control positive group received CCl4 (i.p.), GIII: CCl_4_-rats received 50 mg kg^−1^ vit. E + Se twice a week (i.m.), GIV: CCl_4_-rats received KEE as 250 mg kg^−1^ (p.o.) daily, GV: CCl_4_-rats received KAE as 250 mg kg^−1^ (p.o.) daily and GVI: CCl_4_-rats received KEE + KAE (1:1) as 250 mg kg^−1^ (p.o.) daily. ^a–c^: values with the same superscript letter in the same raw are not significantly different at *p* ≤ 0.05.

**Table 5:** Nephroprotection percentage of *A. hierochuntica* extracts in CCl_4_-induced toxicity in rats.

Parameters	Experimental Groups			
	GIII	GIV	GV	GVI
Creatinine	97.62	73.80	52.38	92.29
Urea	99.85	89.88	97.31	81.58
K	71.53	56.96	40.15	5.11
Total proteins	68.11	77.66	96.73	23.16
Albumin	80.95	63.49	168.25	68.25
MDA	96.23	88.81	37.60	12.90
SOD	59.79	43.34	93.92	77.65
GSH	115.57	80.32	86.89	85.25
* TFP%	100	83.27	97.62	78.85

MDA: malondialdehyde, SOD: superoxide dismutase, GSH: reduced glutathione, * TFP%: total nephroprotection % calculated relatively based on vit. E and Se treatment, GIII: CCl_4_-rats received 50 mg kg^−1^ vit. E + Se twice a week (i.m.), GIV: CCl_4_-rats received KEE as 250 mg kg^−1^ (p.o) daily, GV: CCl_4_-rats received KAE as 250 mg kg^−1^ (p.o.) daily and GVI: CCl_4_-rats received KEE + KAE (1:1) as 250 mg kg^−1^ (p.o.) daily.

**Table 6:** The severity of histopathological alteration in rat kidney’s underlying structure of different experimental groups treated by *A. hierochuntica* extracts.

	GI	GII	GIII	GIV	GV	GVI
Focal inflammatory cells Infiltration between the tubule	−	++	−	−	++	−
Eosinophilic renal cast	−	++	−	−	−	−
Congestion	−	++	+	++	++	++
Focal hemorrhage	−	++	−	−	−	−

+++ = severe, ++ = moderate, + = mild, − = nil, GI: control negative group, GII: control positive group received CCl_4_ (i.p.), GIII: CCl_4_-rats received 50 mg kg^−1^ vit. E + Se twice a week (i.m.), GIV: CCl_4_-rats received KEE as 250 mg kg^−1^ (p.o.) daily, GV: CCl_4_-rats received KAE as 250 mg kg^−1^ (p.o.) daily and GVI: CCl_4_-rats received KEE + KAE (1:1) as 250 mg kg^−1^ (p.o.) daily.

## Data Availability

The data presented in this study are available on request from the corresponding author.

## Abbreviations

ABTS: 2,2’-azino-bis(3-ethylbenzothiazoline-6-sulfonic acid); AOA: antioxidant activity; BHA: butylated hydroxyanisole; BHA: butylated hydroxyanisole; DPPH: 1,1-diphenyl-2-picryl hydrazine; dw: dry weight; GA: gallic acid; GAE: gallic acid equivalent; GSH: reduced-glutathione; HPLC-DAD: high-performance liquid chromatography diode array detection; KAE: *A. hierochuntica* aqueous extract; KEE: *A. hierochuntica* ethanolic extract; MDA: malonaldehyde; QE: quercetin equivalent; RAA: relative antioxidant activity; ROS: reactive oxygen species; RSA: radical scavenging activity; Se: selenium; SE: standard error; SOD: superoxide dismutase; TC: total carotenoids; TC: total carotenoids; TF: total flavonoids; TE: trolox equivalents; TFL: total flavonols; TPC: total phenolic compounds.
